# Routine deworming during antenatal care decreases risk of neonatal mortality and low birthweight: A retrospective cohort of survey data

**DOI:** 10.1371/journal.pntd.0009282

**Published:** 2021-04-29

**Authors:** Bhavneet Walia, Brittany L. Kmush, Sandra D. Lane, Timothy Endy, Antonio Montresor, David A. Larsen

**Affiliations:** 1 Syracuse University Department of Public Health, Syracuse, New York, United States of America; 2 State University of New York Upstate Medical University Department of Infectious Disease, Syracuse, New York, United States of America; 3 Department of Control of Neglected Tropical Diseases, World Health Organization, Geneva, Switzerland; Seoul National University College of Medicine, REPUBLIC OF KOREA

## Abstract

**Background:**

Soil transmitted helminths (STH) are a common infection among pregnant women in areas with poor access to sanitation. Deworming medications are cheap and safe; however, the health benefit of deworming during pregnancy is not clear.

**Methods / Principal findings:**

We created a retrospective cohort of more than 800,000 births from 95 Demographic and Health Survey datasets to estimate the impact of deworming medicine during routine antenatal care (ANC) on neonatal mortality and low birthweight. We first matched births on the probability of receiving deworming during ANC. We then modeled the birth outcomes with the matched group as a random intercept to estimate the effect of deworming during antenatal care after accounting for various risk factors. We also tested for effect modification of soil transmitted helminth prevalence on the impact of deworming during ANC. Receipt of deworming medication during ANC was associated with a 14% reduction in the risk of neonatal mortality (95% confidence interval = 10–17%, n = 797,772 births), with no difference between high and low transmission countries. In low transmission countries, we found an 11% reduction in the odds of low birth weight (95% confidence interval = 8–13%) for women receiving deworming medicine, and in high transmission countries, we found a 2% reduction in the odds of low birthweight (95% confidence interval = 0–5%).

**Conclusions / Significance:**

These results suggest a substantial health benefit for deworming during ANC that may be even greater in countries with low STH transmission.

## Author summary

Soil-transmitted helminths cause a significant burden of disease throughout the world, particularly in communities with limited access to sanitation facilities and clean drinking water. Deworming medicines effectively clear these parasites, are inexpensive, and are well tolerated. However the effectiveness of deworming medicines, particularly for pregnant women, has not been clearly demonstrated. In this paper we analyze more than 800,000 births to measure the effect that deworming medicine during pregnancy has on birth outcomes. When women receive deworming medicine during pregnancy we saw two specific benefits for the baby: first the risk of neonatal mortality (a baby’s death within first 4 weeks of life) decreases by an estimated 14%, and second, the odds of low birthweight were 11% lower in countries with lower transmission of soil-transmitted helminths. In countries with higher transmission of soil-transmitted helminths we saw no effect of deworming medicine on the odds of low birthweight. Given the results, resources leading to widescale distribution of deworming during pregnancy would have a positive effect on child survival and health.

## Introduction

### Background/rationale

Soil transmitted helminths (STH) are parasitic nematodes (worms) that are transmitted by contamination of soil with human feces. The major species of STH that infect humans are *Ascaris lumbricoides* (roundworm) and *Trichuris trichiura* (whipworm), which infect humans via a fecal-oral route, and hookworm (*Ancylostoma duodenale* and *Necator americanus*), whose eggs are shed in fecal matter whereupon hatched larvae then burrow through the skin of the host. STH are estimated to infect more than two billion people across the globe [1], and in 2016 caused the loss of an estimated 3.5 million disability-adjusted life years (DALYs) [2].

STH affect human health in various ways. Hookworm is known to cause iron deficiency and anemia. For pregnant women, the resulting anemia can be particularly severe [3,4]. Infections with *T*. *trichiura* are also likely to cause anemia, and are associated with poor growth and delayed cognitive development in children [5]. Infections with *A*. *lumbricoides* are also associated with poor growth and delayed cognitive development in children [6].

Due to the fecal-oral route of transmission and the life stages in the soil, clean water and adequate sanitation access can easily prevent infections with STH [7–9]. However, sanitation access is still limited in lower-income countries, and therefore periodically clearing the parasites with deworming treatments is a short-term intervention recommended by the WHO in STH endemic areas. The WHO manages a global donation of anthelminthics (albendazole and mebendazole)—with the support of several pharmaceutical companies that donate the medicines—providing them to endemic countries that request them for control programs targeting preschool children and school-age children. The effectiveness of deworming children has been called into question, however, due to recent reviews finding contradictory results of the health impact of mass drug administration in these populations [10–12]. A recent Cochrane review has also found limited evidence that deworming medicine during antenatal impacts birth outcomes or neonatal mortality [13]. However, the review evaluated less than 4,000 pregnancies in four studies, and the authors state that more data are needed to establish the benefit of the intervention or potential lack thereof.

### Objectives

In this study, we explore the connection between anthelminthic treatment of pregnant women during antenatal care and the outcomes of neonatal mortality and low-birth weight using a retrospective cohort of survey data of more than 770,000 births across a broad range of STH transmission settings.

## Methods

### Study design

**Fig 1:**
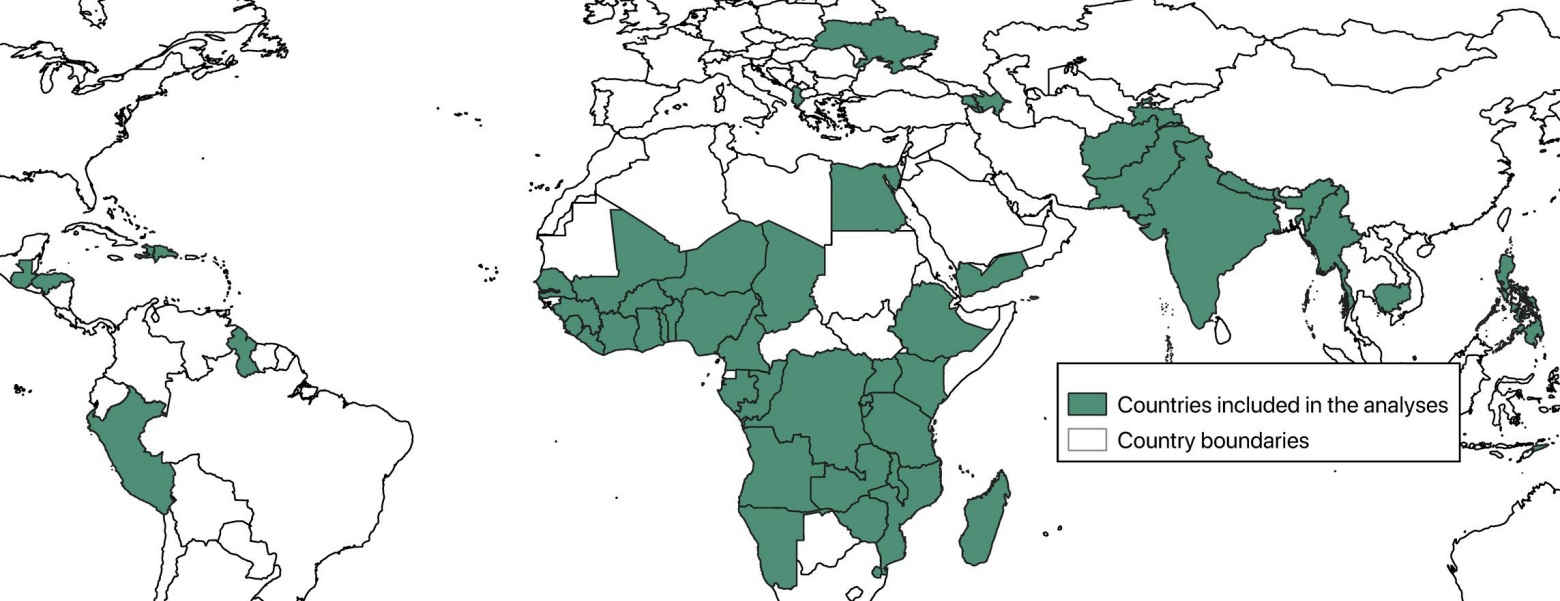
Map of countries that contributed at least one population-based survey to the analysis.

We utilized birth histories from cross-sectional surveys to create a retrospective cohort to measure the impact of routine deworming medicine during antenatal care on subsequent neonatal mortality and low birthweight for births between 1998–2018 in 56 lower income countries (Fig 1).

### Data sources

The Demographic and Health Surveys (DHS) are funded at least in part by the United States Agency for International Development. These surveys utilize nationally representative two-stage cluster samples to generate information on child mortality and women’s fertility. As part of the survey, complete birth histories are recorded for all women aged 15–49 years, including whether or not each child is still alive and the age of the child’s death if the child died. Additionally, information on the most recent pregnancy within the previous 2 years is collected, including various aspects of the woman’s antenatal care (ANC) such as a question on the administration of deworming during the most recent pregnancy. We sought to include all DHS datasets with the following conditions: 1) the survey was conducted in 1990 or after, 2) the survey contained information on deworming during ANC, and 3) the survey was publicly available as of August 23, 2019.

### Outcomes

Neonatal mortality served as the primary outcome. In the DHS questionnaire, the survey respondent classifies the age of their child at death in terms of days, weeks, or months. Significant heaping of neonatal mortality occurs at one month of life in these data. We included children as neonatal deaths if their mother described them dying within the first 28 days of life, the first four weeks of life, or the first month of life.

Low birthweight served as the secondary outcome. In the DHS questionnaire, a child’s weight at birth is included if the child is weighed at birth (approximately 50% of babies in the DHS are weighed at birth). The mother is also asked the child’s perceived birth size. We created a composite indicator of measured low birthweight when available and perceived birth size when measured low birthweight was unavailable. For those children who were weighed at birth, we categorized children as being low birth weight if they were < 2500 grams at birth. For those children who were not weighed at birth, we categorized children as low birth weight if they were perceived to be smaller than average or very small. We also conducted a sensitivity analysis, wherein we limited the low birthweight analyses to those children who were weighed at birth.

### Bias

Better ANC likely acts as a selection bias in women receiving deworming medicines, either through distribution of deworming medicine at ANC itself or for a suggestion from the provider to take deworming medicines. Independent of deworming medicines, women who attend routine antenatal care are predisposed to have better birth outcomes than women who do not attend routine antenatal care. This may include, among other indirect factors such as wealth and education, better ANC and associated improvements in post-natal care. We therefore utilized an exact matching procedure to pre-process the data and reduce the selection bias of receiving deworming medicine [14]. We exactly matched women on their probability of receiving deworming during pregnancy using the MatchIt package in R version 3.6.1 [15,16]. The following equation describes the matching process:

$${Deworming}_{ijk}={ANC}_{ijk}+E_{ijk}+W_{jk}+S_{k}$$

where *Deworming*_*ijk*_ is a dichotomous outcome for woman *i* in household *j* in survey *k*; *ANC*_*ijk*_ is a vector of various aspects of ANC, including attending ANC at any time during pregnancy, being weighed during ANC, being measured during ANC, having blood pressure taken during ANC, giving a urine sample during ANC, giving a blood sample during ANC, receiving intermittent preventive treatment for malaria during ANC, and receiving a neonatal tetanus vaccination during ANC; *E*_*ijk*_ is whether the woman completed primary education or not; *W*_*jk*_ is whether or not house *j* is rich or poor as categorized above or below the median of the asset-based wealth index score; and *S*_*k*_ is a vector of survey characteristics including the proportion of pregnant women receiving deworming medicine as per the survey and the survey dataset.

### Statistical methods

We modeled neonatal mortality as a function of receiving deworming medicine during ANC after adjusting for the following *a priori* determined covariates: the mother’s age (categorized as < 18, 18–35, and >35), the mother’s parity and birth order (categorized as firstborn, 2^nd^ or 3^rd^ born with < 24 months preceding birth interval, 2^nd^ or 3^rd^ born with ≥ 24 months preceding birth interval, 4^th^ or later born with < 24 months preceding birth interval, and 4^th^ or later born with ≥ 24 months preceding birth interval), the presence of a skilled birth attendant during childbirth (doctor, nurse, or midwife), the location of child birth (at a health center or not), the household wealth quintile, the mother’s education (categorized as no education, some primary, or completed primary or higher), the location of the house (urban or rural), the household’s sanitation access (categorized as any or none), the proportion of children aged 1–5 years receiving deworming at the sub-national level as a continuous variable, and the survey dataset as an indicator variable. For three surveys that did not measure deworming in children, we input the median coverage of 0.33. We utilized a Poisson model with the number of days alive (up to 28) included as the exposure and the matched group included as a random intercept. The analysis of neonatal mortality can be described using the following equation:

$$\mu_{ijkl}=e^{ln(t_{ijkl})+\beta_{1}{Deworming}_{ijk}+\chi M_{ijk}+\delta H_{jk}+\kappa S_{k}+\zeta_{l}}$$

$$\zeta_{l}∼N(0,\psi )$$

where *μ_ijkl_* is the rate of death for child *i* in house *j* in survey *k* in matched group *l*, *t_ijkl_* is the number of days the child was alive or at risk of death, *Deworming_ijk_* is whether or not the child’s mother received deworming during ANC, *M_ijk_* is a vector of a child’s mother’s characteristics, *H_jk_* is a vector a child’s household’s characteristics, *S_k_* is a vector of survey characteristics and *ζ_l_* is a random intercept for matched group *l* that is assumed to be normally distributed with a mean of zero. Given uncertainty around date of death and potential heaping at one month of age we also ran a logit model as outlined above but was found to make minimal difference.

We modeled low birth weight as a function of receiving deworming medicine during ANC after adjusting for the same *a priori* determined covariates as previously described. We utilized a logit model with the matched group included as a random intercept. The analysis of low birth weight can be described using the following equation:

$$y_{ijkl}|\pi_{ijkl}∼Binomial(1,\pi_{ijkl})$$

$$logit(\pi_{ijkl})=\beta_{1}{Deworming}_{ijk}+\chi M_{ijk}+\delta H_{jk}+\kappa S_{k}+\zeta_{l}$$

$$\zeta_{l}∼N(0,\psi )$$

where *π_ijkl_* is a dichotomous outcome for child *i* in household *j* in survey *k* in matched group *l*, *Deworming_ijk_* is whether the child’s mother received deworming medicine during ANC, *M_ijk_* is a vector of a child’s mother’s characteristics, *H_jk_* is a vector of a child’s household’s characteristics, *S_k_* is a vector of survey characteristics and *ζ_l_* is a random intercept for matched group *l* that is assumed to be normally distributed with a mean of zero. All analyses were conducted in Stata version 15.1.

We also tested whether the association between deworming medicine and the outcomes of neonatal mortality and low birthweight was moderated by the prevalence of any STH in the country. Based upon estimates provided by Pullan et al. [17], we categorized countries as being low (< 20%) or high (> 20%) STH prevalence and then tested for an interaction between low/high STH prevalence and receiving deworming during ANC using a likelihood ratio test of the log likelihoods.

## Results

### Participants

As of October 2019, a total of 290 survey datasets listed at www.dhsprogram.com contained information on either neonatal mortality, low birthweight, or deworming during ANC. Deworming during ANC was available for 95 of these datasets. One survey dataset (Rwanda 2007–08) did not have measures of low birth weight. This left 95 datasets available to create a retrospective cohort of 825,492 single live births to assess the impact of deworming during ANC on neonatal mortality and 94 datasets available to create a retrospective cohort of 807,957 single live births to assess the impact of deworming during ANC on low birthweight. Following exact matching, 95 datasets and 797,772 women were available for the outcome of neonatal mortality and 94 datasets and 772,155 women were available for the outcome of low birthweight. Fig 1 shows the geographic distribution of countries included in the analyses.

### Descriptive data

Among matched births, 25% of mothers reported receiving deworming medicine during ANC. Two percent of births resulted in a neonatal death (n = 15,784). Only 61% (n = 487,763) of mothers reported a measured birthweight. Among births with a measured birthweight, 12.5% (n = 61,177) were < 2500 grams. Mean birthweight was 3,072 g (standard deviation = 691 g). Among 770,300 births with a perceived birth size, 93,802 mothers (12%) reported the baby to be “smaller than average” and 39,601 mothers reported the baby to be “very small” (5%). Table 1 provides dataset-level information on deworming during ANC and birth outcomes.

**Table 1:** Number of women receiving deworming medicine during routine ANC and birth outcomes.

Survey dataset	Level of STH transmission (Pullan et al.)	Coverage of deworming during ANC	Neonatal deaths without deworming / N without deworming	Neonatal deaths with deworming / N with deworming	LBW without deworming / N without deworming	LBW with deworming / N with deworming
**Afghanistan 2015**	High	3%	396 / 17,773	20 / 616	3,800 / 17,355	136 / 605
**Albania 2008–09**	Low	3%	6 / 933	0 / 31	30 / 930	0 / 31
**Albania 2017–18**	Low	2%	2 / 1952	0 / 45	79 / 1,950	2 / 45
Angola 2015–16*	High	46%	113 / 4,607	69 / 3,939	514 / 4,262	365 / 3,855
**Armenia 2010**	Low	1%	5 / 1,136	0 / 7	70 / 1,134	1 / 7
**Azerbaijan 2006**	Low	3%	31 / 1,430	1 / 51	123 / 1,201	8 / 47
**Benin 2011–12**	Low	76%	44 / 2,066	97 / 6,397	282 / 1,829	766 / 6,205
**Benin 2017–18**	Low	66%	66 / 2,841	122 / 5,578	379 / 2,801	616 / 5,529
**Burkina Faso 2010**	Low	26%	138 / 7,471	32 / 2,562	1,025 / 7,456	299 / 2,559
**Burundi 2010–11**	High	31%	85 / 3,339	29 / 1,1519	445 / 3,281	155 / 1,503
**Burundi 2016–17**	High	66%	31 / 2,888	93 / 5,708	361 / 2,879	582 / 5,697
**Cambodia 2005**	Low	12%	125 / 4,979	9 / 700	740 / 4,940	84 / 692
**Cambodia 2010**	Low	50%	74 / 3,105	45 / 3,106	344 / 2,969	226 / 3,051
**Cambodia 2014**	Low	74%	24 / 1,442	47 / 4,212	157 / 1,428	330 / 4,200
**Cameroon 2011**	High	43%	86 / 4,090	58 / 3,069	579 / 4,059	299 / 3,053
**Chad 2014–15**	Low	23%	185 / 8,076	44 / 2,382	2,362 / 8,011	508 / 2,376
**Comoros 2012**	Low	60%	19 / 722	18 / 1,077	178 / 685	209 / 1,048
**Congo 2011–12**	High	81%	22 / 1,167	71 / 4,888	145 / 1,128	469 / 4,858
**Cote d’Ivoire 2011**	Low	38%	96 / 3,110	43 / 1,877	447 / 3,024	271 / 1,851
**DRC 2013–14**	High	52%	121 / 5,190	107 / 5,724	451 / 5,075	470 / 5,667
**Dominican Republic 2013**	Low	13%	41 / 2,478	4 / 354	345 / 2,472	46 / 354
**Egypt 2014**	Low	4%	85 / 9,981	3 / 397	1,413 / 9,968	51 / 393
**Eswatini 2006–07**	High	12%	37 / 1,583	5 / 212	121 / 1,557	15 / 207
**Ethiopia 2011**	High	6%	176 / 7,099	15 / 422	2,283 / 7081	120 / 422
**Ethiopia 2016**	High	6%	149 / 6,438	10 / 415	1,668 / 6,366	95 / 411
**Gabon 2012**	High	64%	27 / 1,416	44 / 2,495	218 / 1,302	323 / 2,434
**Gambia 2013**	High	43%	38 / 2,947	27 / 2,215	541 / 2,939	300 / 2,211
**Ghana 2008**	Low	40%	28 / 1,139	14 / 746	136 / 1,130	90 / 743
**Ghana 2014**	Low	44%	41 / 2,280	31 / 1,800	1,203 / 8,431	89 / 623
**Guatemala 2014–15**	High	7%	110 / 8,438	8 / 623	1,203 / 8,431	89 / 623
**Guinea 2012**	High	30%	88 / 3,368	23 / 1,473	387 / 3,361	131 / 1,473
**Guyana 2009**	High	20%	17 / 1,135	4 / 289	175 / 1,127	41 / 285
**Haiti 2005–06**	Low	7%	74 / 3,566	9 / 286	1,083 / 3,566	78 / 286
**Haiti 2012**	Low	15%	104 / 4,294	16 / 785	1,331 / 4,288	199 / 785
**Haiti 2016–17**	Low	10%	100 / 4,102	7 / 477	991 / 4,102	99 / 477
**Honduras 2005–06**	High	8%	78 / 6,632	11 / 547	1,101 / 6,625	108 / 544
**Honduras 2011–12**	High	7%	92 / 7,687	12 / 612	1,069 / 7,681	97 / 612
**India 2005–06**	High	4%	782 / 34,248	26 / 1,433	7,304 / 33,761	284 / 1,422
**India 2015–16**	High	15%	3,504 / 159,219	468 / 29,185	25,971 / 155,748	4,785 / 28,940
**Kenya 2008**	Low	20%	57 / 2,770	13 / 672	348 / 2,747	54 / 668
**Kenya 2014**	Low	31%	66 / 4,744	41 / 2,179	549 / 4,679	202 / 2,172
**Liberia 2007**	High	30%	62 / 2,600	20 / 1,108	539 / 2,592	180 / 1,106
**Liberia 2013**	High	57%	55 / 2,230	61 / 2,978	459 / 2,228	543 / 2,971
**Madagascar 2008–09**	High	40%	93 / 4,984	47 / 3,373	916 / 4,911	531 / 3,320
**Malawi 2010**	Low	28%	209 / 9,518	78 / 3,759	1,176 / 9,375	469 / 3,706
**Malawi 2015–16**	Low	52%	123 / 6,289	115 / 6,816	780 / 6,245	841 / 6,781
**Maldives 2009**	Low	18%	17 / 2,482	0 / 552	261 / 2,481	61 / 552
**Mali 2012–13**	Low	29%	120 / 4,461	42 / 1,847	732 / 4,225	225 / 1,818
**Mali 2018**	Low	49%	83 / 3,108	60 / 2,941	524 / 2,787	571 / 2,875
**Mozambique 2011**	High	33%	137 / 4,804	66 / 2,390	593 / 4,594	286 / 2,352
**Myanmar 2015–16**	High	57%	33 / 1,614	32 / 2,127	214 / 1,531	223 / 2,079
**Namibia 2006–07**	Low	8%	58 / 3,293	6 / 289	460 / 3,261	48 / 281
**Namibia 2013**	Low	7%	52 / 3,337	4 / 265	439 / 3,310	34 / 262
**Nepal 2006**	High	19%	80 / 3,276	15 / 786	639 / 3,274	139 / 786
**Nepal 2011**	High	59%	33 / 1,629	42 / 2,353	302 / 1,627	379 / 2,352
**Nepal 2016**	High	74%	21 / 1,021	28 / 2,910	163 / 1,020	402 / 2,905
**Niger 2012**	Low	51%	64 / 3,705	65 / 3,793	854 / 3,490	810 / 3,748
**Nigeria 2008**	High	10%	440 / 15,188	36 / 1,656	2,379 / 15,062	172 / 1,650
**Nigeria 2013**	High	16%	465 / 16,073	91 / 3,001	2,445 / 15,971	315 / 2,991
**Pakistan 2012–13**	Low	2%	235 / 6,136	5 / 156	1,340 / 6,123	38 / 156
**Pakistan 2017–18**	Low	2%	194 / 6,823	2 / 163	1,344 / 6,804	32 / 159
**Peru 2004–08**	High	4%	79 / 8,864	1 / 348	898 / 8,858	38 / 348
**Peru 2009**	High	3%	72 / 7,871	1 / 280	750 / 7,866	30 / 280
**Peru 2010**	High	3%	59 / 7,056	1 / 253	687 / 7,055	32 / 253
**Peru 2011**	High	3%	60 / 6,967	2 / 251	646 / 6,966	28 / 251
**Peru 2012**	High	3%	60 / 6,887	1 / 243	576 / 6,84	28 / 243
**Philippines 2008**	High	5%	45 / 4,011	3 / 196	823 / 3,999	54 / 196
**Philippines 2013**	High	5%	44 / 4,571	5 / 263	782 / 3,719	48 / 214
**Philippines 2017**	High	7%	85 / 7,016	5 / 498	837 / 5,939	68 / 423
**Rwanda 2007–08**	High	19%	43 / 2,567	9 / 617	.	.
**Rwanda 2010**	High	40%	62 / 3,754	34 / 2,453	348 / 3,742	178 / 2,443
**Rwanda 2014–15**	High	50%	44 / 2,934	37 / 2,945	217 / 2,923	179 / 2,934
**STP 2008**	Low	59%	6 / 551	7 / 794	45 / 537	52 / 770
**Senegal 2010–11**	Low	25%	141 / 5,656	51 / 1,927	1,143 / 5,641	388 / 1,922
**Senegal 2012–13**	Low	28%	59 / 2,823	17 / 1,107	697 / 2,821	236 / 1,105
**Senegal 2014**	Low	30%	110 / 5,664	32 / 2,411	1,406 / 5,662	512 / 2,409
**Senegal 2015**	Low	32%	54 / 2,903	31 / 1,369	672 / 2,902	234 / 1,368
**Senegal 2016**	Low	35%	66 / 2,698	19 / 1,466	642 / 2,697	241 / 1,462
**Senegal 2017**	Low	44%	107 / 4,290	72 / 3,380	891 / 4,286	670 / 3,374
**Sierra Leone 2008**	High	47%	61 / 1,945	57 / 1,720	325 / 1,913	283 / 1,687
**Sierra Leone 2013**	High	74%	79 / 2,110	198 / 6,140	326 / 2,050	675 / 6,038
**Tajikistan 2017**	Low	2%	45 / 4,000	0 / 83	296 / 3,867	10 / 79
**Tanzania 2015–16**	High	63%	47 / 2,563	74 / 4,306	273 / 2,542	362 / 4,284
**Timor-Leste 2009–10**	High	14%	97 / 4,918	10 / 832	727 / 4,813	110 / 831
**Timor-Leste 2016**	High	18%	61 / 3,931	19 / 840	358 / 3252	60 / 763
**Togo 2013–14**	Low	62%	38 / 1,807	57 / 2,920	287 / 1,789	292 / 2,904
**Uganda 2006**	Low	27%	63 / 3,464	23 / 1,293	648 / 3,435	203 / 1,289
**Uganda 2011**	Low	51%	69 / 2,326	39 / 2,375	414 / 2,281	324 / 2,321
**Uganda 2016**	Low	61%	112 / 3,888	110 / 6,129	574 / 3,800	692 / 6,050
**Ukraine 2007**	Low	7%	4 / 960	1 / 75	33 / 960	7 / 75
**Yemen 2013**	High	3%	174 / 9,776	8 / 352	3,109 / 9,756	137 / 351
**Zambia 2007**	High	39%	69 / 2,293	31 / 1,450	237 / 2,273	109 / 1,444
**Zambia 2013**	High	66%	56 / 3,038	84 / 6,004	335 / 3,008	527 / 5,941
**Zimbabwe 2010–11**	High	3%	59 / 3,211	1 / 103	279 / 3,164	10 / 103
**Zimbabwe 2015**	High	3%	63 / 4,152	3 / 145	391 / 4,145	9 / 145

### Main results

Before adjusting for any other covariates, 2.1% of births where mothers did not receive deworming during ANC (12,873 / 610,118) and 1.8% of births where mothers did receive deworming (3,622 / 202,501) during ANC died within the neonatal period (relative risk of cumulative incidence = 0.85). Before adjusting for any other covariates, 16.9% of babies where mothers did not receive deworming during ANC (102,802 / 505,383) and 13.3% babies where mothers did receive deworming during ANC (26,590 / 199,772) were considered low birth weight (relative risk of cumulative incidence = 0.79).

After adjusting for selection bias via exact matching and including other factors hypothesized to be associated with neonatal mortality, receiving deworming during routine ANC was associated with a 14% reduction in the risk of neonatal mortality (IRR = 0.86, 95% CI = 0.83–0.90) (Table 2). This relationship was not moderated by STH prevalence: the likelihood ratio test for interaction was not statistically significant (likelihood ratio [LR] = 2.31, p = 0.128).

**Table 2:** Results from Poisson regression analysis of deworming during ANC and neonatal mortality.

Measure	Categorization	Unadjusted IRR (95% CI)	P-value	Adjusted IRR (95% CI)	P-value
**Deworming**	No deworming during ANC	Ref.	Ref.	Ref.	Ref.
	Deworming during ANC	0.81 (0.78–0.85)	< 0.001	0.86 (0.83–0.90)	< 0.001
**Skilled birth attendant**	No doctor, nurse, or midwife during delivery	Ref.	Ref.	Ref.	Ref.
	Doctor, nurse, or midwife during delivery	0.91 (0.88–0.94)	< 0.001	1.01 (0.94–1.08)	0.736
**Facility delivery**	Child was delivered somewhere other than a health center	Ref.	Ref.	Ref.	Ref.
	Child was delivered at a health center	0.92 (0.89–0.95)	< 0.001	1.11 (1.0–4–1.19)	0.002
**Mother’s age at delivery**	18–35 years old	Ref.	Ref.	Ref.	Ref.
	< 20 years old	1.31 (1.25–1.38)	< 0.001	1.17 (1.11–1.24)	< 0.001
	> 35 years old	1.59 (1.52–1.66)	< 0.001	1.59 (1.52–1.66)	< 0.001
**Mother’s parity**	Child is firstborn	Ref.	Ref.	Ref.	Ref.
	2^nd^ born ≥ 24 month preceding birth interval	0.67 (0.64–0.71)	< 0.001	0.69 (0.65–0.73)	< 0.001
	2^nd^ born < 24 month preceding birth interval	0.91 (0.85–0.98)	0.013	0.91 (0.85–0.98)	0.015
	3^rd^ born or later ≥ 24 month preceding birth interval	0.90 (0.86–0.93)	< 0.001	0.78 (0.74–0.82)	< 0.001
	3^rd^ born or later < 24 month preceding birth interval	1.45 (1.38–1.52)	< 0.001	1.30 (1.23–1.38)	< 0.001
**Mother’s education**	No education	Ref.	Ref.	Ref.	Ref.
	Some primary school	0.95 (0.90–0.99)	0.022	1.03 (0.98–1.08)	0.302
	Completed primary school	0.93 (0.88–0.99)	0.020	1.03 (0.96–1.11)	0.367
	Some secondary school or higher	0.71 (0.68–0.74)	< 0.001	0.88 (0.83–0.94)	< 0.001
**Household location**	Rural	Ref.	Ref.	Ref.	Ref.
	Urban	1.14 (1.10–1.18)	< 0.001	0.97 (0.93–1.01)	0.168
**Sanitation access**	Any type of sanitation access	Ref.	Ref.	Ref.	Ref.
	No sanitation access	1.29 (1.24–1.33)	< 0.001	1.13 (1.08–1.18)	< 0.001
**Household wealth**	Poorest wealth quintile	Ref.	Ref.	Ref.	Ref.
	Poorer wealth quintile	0.98 (0.94–1.03)	0.403	1.03 (0.99–1.08)	0.152
	Middle wealth quintile	0.90 (0.86–0.94)	< 0.001	1.05 (0.99–1.10)	0.113
	Richer wealth quintile	0.82 (0.78–0.86)	< 0.001	1.01 (0.95–1.08)	0.776
	Richest wealth quintile	0.69 (0.66–0.73)	< 0.001	0.92 (0.85–0.99)	0.023
**Deworming coverage**	Proportion of children aged 1–5 receiving deworming (continuous)	0.51 (0.44–0.60)	< 0.001	0.71 (0.83–0.90)	< 0.001

N = 800,351 children, 6,870 matched groups (adjusted model only), 95 survey datasets ranging from year 2005–2018. Unadjusted model standard errors were adjusted for survey cluster and included survey dataset as a covariate. Adjusted model included a random intercept of matched group and survey dataset as a covariate. Ref. refers to the reference category for the variable in the regression model.

After adjusting for selection bias via exact matching and including other factors hypothesized to be associated with low birthweight, receiving deworming during routine ANC was associated with a 6% reduction in the odds of low birthweight (Odds ratio [OR] = 0.94, 95% CI = 0.92–0.96). This relationship was moderated by STH prevalence (LR = 24.42, p < 0.001). In low transmission countries (< 20% national STH prevalence according to Pullan et al. [17]), deworming during routine ANC was associated with an 11% reduction in the odds of low birthweight (OR = 0.89, 95% CI = 0.87–0.92). In high transmission countries (> 20% national STH prevalence), deworming during routine ANC was associated with a 2% reduction in the odds of low birthweight (OR = 0.98, 95% CI = 0.95–1.00). Table 3 shows the results from the unadjusted and adjusted analyses of low birthweight.

**Table 3:** Results from logistic regression analysis of deworming during ANC and low birthweight.

Measure	Categorization	Unadjusted OR (95% CI)	P-value	Adjusted OR (95% CI)	P-value
**Deworming**	No deworming during ANC and < 20% national prevalence	Ref.	Ref.	Ref.	Ref.
	Deworming during ANC and < 20% national prevalence	0.80 (0.78–0.83)	< 0.001	0.89 (0.87–0.92)	< 0.001
	No deworming during ANC and > 20% national prevalence	Ref.	Ref.	Ref.	Ref.
	Deworming during ANC and > 20% national prevalence	0.89 (0.87–0.91)	< 0.001	0.98 (0.95–1.00)	0.046
**Skilled birth attendant**	No doctor, nurse, or midwife during delivery	Ref.	Ref.	Ref.	Ref.
	Doctor, nurse, or midwife during delivery	0.68 (0.67–0.69)	< 0.001	0.85 (0.83–0.88)	< 0.001
**Facility delivery**	Child was delivered somewhere other than a health center	Ref.	Ref.	Ref.	Ref.
	Child was delivered at a health center	0.69 (0.68–0.71)	< 0.001	0.93 (0.91–0.95)	< 0.001
**Mother’s age at delivery**	18–35 years old	Ref.	Ref.	Ref.	Ref.
	< 20 years old	1.30 (1.27–1.32)	< 0.001	1.15 (1.12–1.17)	< 0.001
	> 35 years old	1.08 (1.06–1.11)	< 0.001	1.05 (1.02–1.07)	< 0.001
**Mother’s parity**	Child is firstborn	Ref.	Ref.	Ref.	Ref.
	2^nd^ born ≥ 24 month preceding birth interval	0.83 (0.81–0.84)	< 0.001	0.83 (0.81–0.84)	< 0.001
	2^nd^ born < 24 month preceding birth interval	0.94 (0.92–0.97)	< 0.001	0.90 (0.87–0.92)	< 0.001
	3^rd^ born or later ≥ 24 month preceding birth interval	0.91 (0.89–0.92)	< 0.001	0.80 (0.79–0.82)	< 0.001
	3^rd^ born or later < 24 month preceding birth interval	0.99 (0.97–1.01)	0.372	0.86 (0.84–0.88)	< 0.001
**Mother’s education**	No education	Ref.	Ref.	Ref.	Ref.
	Some primary school	0.89 (0.87–0.91)	< 0.001	0.90 (0.88–0.92)	< 0.001
	Completed primary school	0.77 (0.75–0.79)	< 0.001	0.80 (0.77–0.82)	< 0.001
	Some secondary school or higher	0.67	< 0.001	0.73 (0.71–0.75)	< 0.001
**Household location**	Rural	Ref.	Ref.	Ref.	Ref.
	Urban	1.26 (1.24–1.28)	< 0.001	0.95 (0.94–0.97)	< 0.001
**Sanitation access**	Any type of sanitation access	Ref.	Ref.	Ref.	Ref.
	No sanitation access	1.32 (1.30–1.34)	< 0.001	1.10 (1.08–1.12)	< 0.001
**Household wealth**	Poorest wealth quintile	Ref.	Ref.	Ref.	Ref.
	Poorer wealth quintile	0.83 (0.82–0.85)	< 0.001	0.90 (0.88–0.92)	< 0.001
	Middle wealth quintile	0.74 (0.73–0.76)	< 0.001	0.83 (0.81–0.85)	< 0.001
	Richer wealth quintile	0.68 (0.67–0.70)	< 0.001	0.81 (0.78–0.83)	< 0.001
	Richest wealth quintile	0.56 (0.55–0.58)	< 0.001	0.72 (0.69–0.74)	< 0.001
**Deworming coverage**	Proportion of pregnant women receiving deworming (continuous)	0.71 (0.66–0.76)	< 0.001	0.82 (0.77–0.88)	< 0.001

N = 779,790 children, 6,771 matched groups (adjusted model only), 94 survey datasets ranging from year 2005–2018. Unadjusted model standard errors were adjusted for survey cluster and included survey dataset as a covariate. Adjusted model included a random intercept of matched group and survey dataset as a covariate. Ref. refers to the reference category for the variable in the regression model.

Among the children who were weighed at birth, deworming during ANC was not globally associated with low birthweight (OR = 0.98, 95% CI = 0.96–1.00, p = 0.109). There was a significant interaction between STH prevalence and receipt of deworming medicine during ANC (LR 11.08, p < 0.001). Deworming during ANC effectively reduced the odds of low birth weight in low transmission countries (OR = 0.93, 95% CI = 0.90–0.97), but not in high transmission countries (OR = 1.00, 95% CI = 0.98–1.04).

## Discussion

### Key results

We provide empirical evidence as to the impact of deworming in pregnant women on the neonatal health of their infants (low birthweight and neonatal mortality) across a large range of STH endemic countries. Using a retrospective birth cohort of more than 770,000 births, we find that children born to mothers who received deworming during antenatal care have a 14% lower risk of neonatal mortality (95% CI = 11–16%). In countries with < 20% estimated national prevalence of any STH, deworming during antenatal care was associated with an 11% reduction in the odds of low birthweight (95% CI = 8–13%). In countries with > 20% estimated national prevalence of any STH, deworming during ANC was associated with a 3% reduction in the odds of low birthweight (95% CI = 1–5%).

### Limitations

A number of limitations are present in this analysis. First, there is selection bias in women receiving deworming during ANC. Due to the inequity observed in children receiving deworming medicines [18], we suspect that mothers receiving deworming during ANC are predisposed to have better birth outcomes than mothers not receiving deworming during ANC. We have attempted to mitigate the selection bias by matching mothers on the probability that they receive deworming during ANC before analysis. There may still be some residual confounding in this analysis. Second, this study relies on mothers’ recall of medicines received during their most recent pregnancy. There may be some recall error associated with mothers’ abilities to remember all the medicines and supplements they received during pregnancy, and recall bias may be present if mothers of babies suffering neonatal deaths are more likely remember receiving deworming medicine or not better than mothers of surviving babies. We have attempted to mitigate this limitation by only including the most recent pregnancy and only including pregnancies within 5 years of the study. There may also be recall bias if mothers in lower transmission settings are less aware of the need for deworming medicines and thus less likely to recall receiving the medicines during ANC. Third, because this study creates a retrospective cohort from historical birth outcomes, we are unable to account for the mother’s or child’s health status at the time of pregnancy and birth. Fourth, this study relies on mothers’ recall of the age of the child at death, which may lead to misclassification error in our outcome of neonatal mortality. As such, we observed heaping of death at one month of age. We have chosen to include children dying at one month of age as neonatal deaths. We do not expect the misclassification error to be related to receiving deworming during ANC and suspect no bias in this regard. Fifth, our measure of low birthweight is a composite indicator of a mother’s perception of birth size or a mother’s recall of the child’s birth weight if the child was weighed at birth. This approach may lead to misclassification error in our outcome of low birth weight, but we do not expect it to bias the results, as it is not likely to be associated with receiving deworming medicine during ANC. There were also no inferential differences in the results when we limited the analysis to those babies who were weighed at birth. And sixth, the type of deworming drug taken by the mother is not recorded which raises a potential non-STH benefit. Mebendazole (500 mg) or albendazole (400 mg) are the most commonly used deworming drugs in these countries, and are most likely to be the drug taken during pregnancy in this cohort of women. These two benzimidazoles are poorly absorbed in the intestine and have no documented efficacy on virus nor bacteria. In addition, with the exception of enterobiasis, the efficacy against other helminthiasis is poor at the dose provided. We think therefore that there are no relevant “off-target” effects for the drugs taken during pregnancy.

### Interpretation

These results suggest a strong benefit of deworming during ANC in both high and low transmission areas, particularly for the outcome of neonatal mortality. Perplexingly, the effect of deworming during ANC on low birthweight was greater in low transmission areas than in high transmission areas. We cannot explain this result, but humbly suggest some potential mechanisms. In high transmission settings school-aged children are dewormed more often. The more frequent deworming could potentially reduce worm burdens for adult women. However, in high transmission areas individual worm burdens are likely to be greater in high transmission areas, potentially causing greater damage in terms of anemia and other effects before deworming medicine is taken. Finally, reinfection occurs rapidly in areas of high transmission, particularly among those who were previously infected [19,20]. The half-life of deworming medicines is quite short (< 24 hours), and very little chemoprophylaxis is provided for women treated. Regardless of the mechanism driving the observed relationship, we consider that due to reinfection, a permanent solution of the problem caused by STH can only be obtained by a substantial improvement in the access to improved sanitation. However, since this process of sanitation improvement is normally slow and expensive, periodic deworming should be available to all pregnant women in STH-endemic countries.

Periodic deworming is a very low-cost intervention. Where infrastructure for distribution is in place, such as through school programs, vaccination campaigns or routine ANC, the intervention cost is a few cents for each individual treated [21,22]. Considered safe during pregnancy, the risk of side effects from the drug administration is also minimal because benzimidazoles are poorly absorbed and are normally expelled after killing the worms present in the intestine [23]. These results provide impetus to improve access to deworming medicines during routine ANC.

While some have called for randomized trials of deworming for pregnant women to establish efficacy, these results suggest that deworming reduces the risk of neonatal mortality and low birthweight. To our knowledge this is the largest analysis conducted on deworming and birth outcomes (more than 770,000 births–previous Cocherane review has < 3,400), and in our view is the more appropriate approach to provide evidence on a relatively rare event like neonatal mortality. From an empirical perspective, a sample size of 3,400 does not allow for a great deal of variation in the outcome variable. That is, for low probability (*p*) events, *np* is typically low unless *n* is sufficiently large to compensate. The approach we used herein also avoids the ethical dilemma of withholding an intervention from the control group, the major limitation to conducting randomized control trials of clearing parasites with deworming medicine.

The WHO recommends periodic deworming of children and women of reproductive age (including pregnant women after the first trimester) [23]. While in the last 10 years the coverage of the intervention scaled up significantly for preschool and school age children, reaching over 65% in 2017 [18,24], the scale up of coverage for women of reproductive age has been much slower, with an average of 23% of pregnant women in STH endemic countries receiving deworming during ANC but higher coverage in African countries (mean 35%) [24,25]. A recent meeting of the WHO Advisory Group on deworming in girls and women of reproductive age [26] urged all stakeholders in women’s health to take immediate action in their respective domains to ensure that women of reproductive age are now included in their STH policies and programs, and to invite WHO to develop support material to facilitate implementation of deworming programs [27].

The traditional understanding of host-parasite interactions during pregnancy is that the parasite takes nutrients from the pregnant host, that either causes anemia and/or reduces the nutrients passing to the fetus leading to intrauterine growth restriction [28,29], increased risk of low birthweight, and then subsequent neonatal mortality [30]. In this analysis, the impact of deworming on low birthweight was quite modest in high transmission areas (3% reduction) compared to the impact of deworming on neonatal mortality (14% reduction). These results provide some evidence that helminth infections may indeed decrease nutrient flow to the fetus, but perhaps raise questions about other ways in which helminth infections affect pregnancy.

We suspect the primary mechanism of protection for deworming medicines to be through improving the health of the mother and subsequently the health of the fetus. In order to survive within their hosts, STH modify their hosts’ immune responses, downregulating T-cell activity and other immune responses [31]. This immunosuppression affects not only the hosts’ immune response to the STH, but also the immune response to other pathogens. For example, chronic STH infection is associated with suboptimal immunity following the receipt of various vaccines [32]. And, in areas of high prevalence of STH, routine deworming led to improved immune responses to non-STH pathogens [33]. This effect of compromising maternal immunity may be particularly serious given that a pregnant woman’s immune response is downregulated by the 18^th^ week of fetal gestation to ensure the mother’s body does not reject the fetus as a foreign body [34,35]. Furthermore, immune system downregulation passes through to the fetus. Helminth infections prime the immune system of the fetus, making children born to mothers with helminth infections more vulnerable to not only helminth infections after they are born, but other pathogens as well [36,37]. This mechanism could lead to increased risk of neonatal mortality for children of women who do not receive deworming medicines during antenatal care.

## Conclusion

This large retrospective cohort of survey data suggests that deworming during antenatal care is associated with decreased neonatal mortality and low birthweight. This protection may even be greater in countries with lower STH transmission.
